# *Dictyophora indusiata* and *Bacillus aryabhattai* improve sugarcane yield by endogenously associating with the root and regulating flavonoid metabolism

**DOI:** 10.3389/fpls.2024.1326917

**Published:** 2024-03-07

**Authors:** Mingzheng Duan, Xiang Li, Xiaojian Wu, Shengfeng Long, Hairong Huang, Yijie Li, Qi-Huai Liu, Guanghu Zhu, Bin Feng, Sunqian Qin, Changning Li, Hai Yang, Jie Qin, Zhendong Chen, Zeping Wang

**Affiliations:** ^1^ Guangxi Academy of Agricultural Sciences, Nanning, China; ^2^ Yunnan Key Laboratory of Gastrodia Elata and Fungal Symbiotic Biology, College of Agronomy and Life Sciences, Zhaotong University, Zhaotong, China; ^3^ Center for Applied Mathematics of Guangxi (GUET), Guilin, China; ^4^ Laibin Academy of Agricultural Sciences, Laibin, China

**Keywords:** *Bacillus*, full-length 16S rRNA, metabolome, metabarcoding, plant growth, sugarcane root

## Abstract

**Introduction:**

Endophytes play a significant role in regulating plant root development and facilitating nutrient solubilization and transportation. This association could improve plant growth. The present study has uncovered a distinct phenotype, which we refer to as "white root", arising from the intricate interactions between endophytic fungi and bacteria with the roots in a sugarcane and bamboo fungus (*Dictyophora indusiata*) intercropping system.

**Methods:**

We investigated the mechanisms underlying the formation of this “white root” phenotype and its impact on sugarcane yield and metabolism by metabarcoding and metabolome analysis.

**Results and Discussion:**

Initial analysis revealed that intercropping with *D. indusiata* increased sugarcane yield by enhancing the number of viable tillers compared with bagasse and no input control. Metabarcoding based on second-generation and third-generation sequencing indicated that *D. indusiate* and *Bacillus aryabhattai* dominates the fungal and bacterial composition in the “white root” phenotype of sugarcane root. The coexistence of *D. indusiata* and *B. aryabhattai* as endophytes induced plant growth-promoting metabolites in the sugarcane root system, such as lysoPC 18:1 and dihydrobenzofuran, probably contributing to increased sugarcane yield. Furthermore, the association also enhanced the metabolism of compounds, such as naringenin-7-O-glucoside (Prunin), naringenin-7-O-neohesperidoside (Naringin)*, hesperetin-7-O-neohesperidoside (Neohesperidin), epicatechin, and aromadendrin (Dihydrokaempferol), involved in flavonoid metabolism during the formation of the endophytic phenotype in the sugarcane root system. These observations suggest that the “white root” phenotype promotes sugarcane growth by activating flavonoid metabolism. This study reports an interesting phenomenon where *D. indusiata*, coordinate with the specific bacteria invade, forms a “white root” phenotype with sugarcane root. The study also provides new insights into using *D. indusiata* as a soil inoculant for promoting sugarcane growth and proposes a new approach for improve sugarcane cultivation.

## 1 Introduction

Plants live in nature alongside various microbes, including archaea, bacteria, fungi, and protists. These microorganisms, collectively known as the plant microbiota, form intricate communities and play a significant role in regulating the growth and productivity of plants (Hassani et al., 2018). Endophytes, which primarily reside within plant tissues, play a significant role in regulating plant root development and facilitating nutrient solubilization and transportation (Verma et al., 2021; Santoyo et al., 2016). Therefore, gaining a deeper understanding of the relationship of interaction between endophytes and plants is significant for optimizing agricultural production and ecological environments.

Sugarcane is the world’s largest sugar crop and is extensively cultivated in the Guangxi Province of China. Utilizing sugarcane bagasse for field incorporation and growing *Dictyophora indusiata* (bamboo fungus) as intercrops have helped enhance the added value of the sugarcane industry. Therefore, we conducted an experiment intercropping sugarcane and *D. indusiata* from March to October 2022. The studies have reported that the intercropping system, *D. indusiata* and sugarcane, enhances soil chemical properties and activates soil metabolism (Duan et al., 2023a); Additionally, the same genomic and metabolomic study revealed that the fruiting body of *D. indusiata* could synthesize IAA (indole-3-acetic acid) (Duan et al., 2023b), which probably leads to improved soil fertility and sugarcane yield. These preliminary studies suggest that intercropping *D. indusiata* with sugarcane benefits both species.

**Figure 1:**
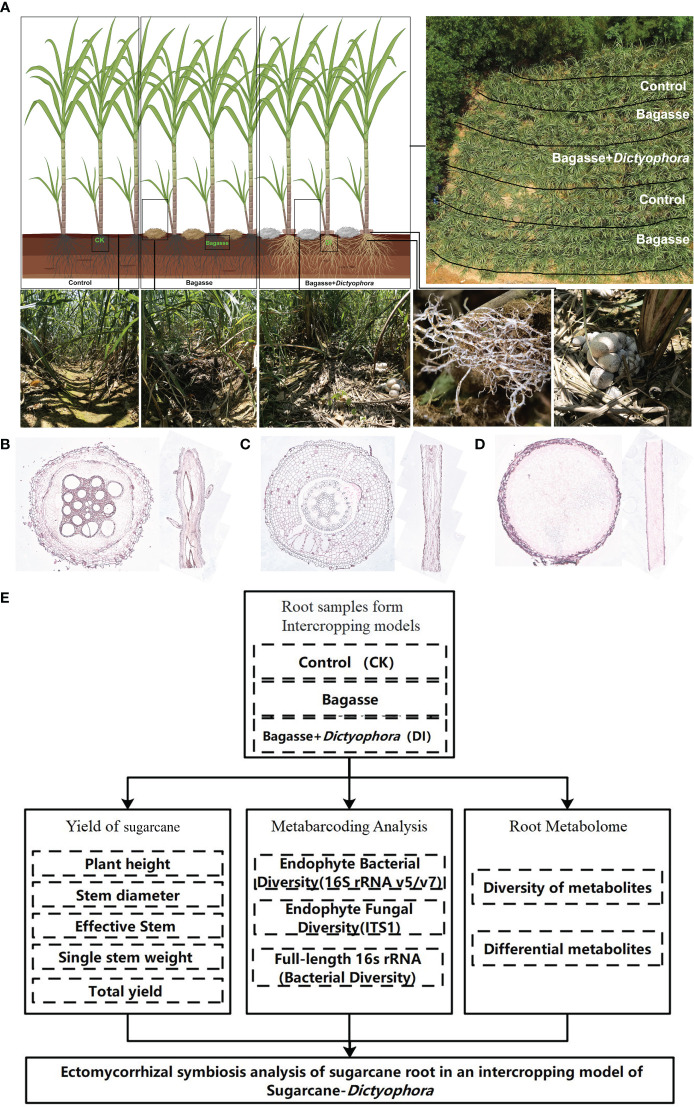
The experimental approach used in our study. **(A)** Sugarcane and *Dictyophora indusiata* intercropping. The image in the upper right shows an aerial view of the field and the division of the treatments within the field. The three images in the lower left show the sugarcane rows corresponding to the different treatments (CK, Bagasse and DI), and the two images in the lower right show the growth of mycelium under the effects of the DI on the sugarcane root system. **(B)** Microscopic anatomical figure of sugarcane root under CK treatments. **(C)** Microscopic anatomical figure of sugarcane root under Bagasse treatments. **(D)** Microscopic anatomical figure of sugarcane root under DI treatments. **(E)** The technical route of this study.

We further investigated the interaction between the sugarcane root system and *D. indusiata* during intercropping to build upon the aforementioned experiment. This approach has led to the discovery of a fascinating phenotype we named as “white root” (**Figure 1A**), wherein the sugarcane root system forms a symbiotic relationship with the *D. indusiata* mycelium in the intercropping system. Notably, the mycelium was found to be enriched inside the root system (**Figure 1D**). Through this study, we have unveiled the unique interaction and mutualism between sugarcane and fungi. As a result, we have identified an interesting interacting relationship between *D. indusiata* and sugarcane roots, which has the potential to enhance sugarcane yield. Overall, DI has the potential to be developed as a novel microbial inoculant, and the development of microbial inoculants can enhance crop yield and resistance to diseases in an environmentally friendly manner (Kashyap et al., 2023). Therefore, elucidating the mechanism underlying this association will provide new insights for sugarcane cultivation and yield improvement.

Metabarcoding and metabolomics are the rapidly developing omics technologies that help reveal the mechanisms underlying plant-microbe interactions. A study based on metabarcoding and metabolome techniques revealed the mechanisms promoting plant growth by a fairy ring fungi (Duan et al., 2022b). Similar metabarcoding and metabolome techniques will unravel the interaction mechanisms between *D. indusiata* and sugarcane roots. Especially with the third-generation sequencing technology-based full-length sequencing of metabarcoding tags, it is possible to accurately obtain longer microbial barcode sequences as mentioned by Rubiola, full-length 16S rRNA sequencing offers several advantages in identifying the dominant bacteria within microbial communities (Rubiola et al., 2022).

Therefore, the present study aimed to unravel the interactions between endophytes and metabolism between *D. indusiata* and sugarcane roots and the impact of this interaction on sugarcane yield. We analyzed the effects of cultivating *D. indusiata* on sugarcane yield using a blank (CK) and bagasse (non-inoculated) as controls. Then, the relationship of endophytes and metabolism involved in the “white root” phenotype were analyzed using metabarcoding and metabolome techniques. Finally, through correlation analysis, we elucidated the regulatory potential of key metabolites of major fungi and bacteria during the interactions between endophytes and metabolism.

## 2 Materials and methods

### 2.1 Materials

The experiment took place in a cultivated land in Laibin City, Guangxi Province, China (23°16′N, 108°24′E) from February to August 2022. The following treatments were used: control (CK), standard management conditions for sugarcane growth; Bagasse, crushed and air-dried bagasse applied to the soil between rows of sugarcane plants; and DI, crushed and air-dried bagasse mixed with *D. indusiata* (DI) strains obtained via wheat grain fermentation, applied to the soil between rows of sugarcane plants (**Figure 1A**). The local sugarcane variety GT42 was utilized in this study. The sugarcane plants were initially grown with an interrow spacing of approximately 50 cm and a row depth of 30 cm. After 60 days of cultivation, when the sugarcane plants reached a height of approximately 50 cm, five semi-rectangular areas were marked for the three treatments (**Figure 1A**). Soil moisture levels were maintained at a high level throughout the experiment. After approximately 90 days, the fruiting bodies of DI matured. Root samples were randomly collected from the middle three treatments in the rows, with three biological replicates per treatment. Using a microscope (Axio Imager M2m, ZEISS, Germany) and resorcinol staining method (Resorcinol staining solution, No. G1060, Servicebio, China), the internal microstructures of the root systems from three treatments were observed. It can be seen that there were no fungal mycelium observed in the cross-section and longitudinal section of the root systems from treatments CK (**Figure 1B**) and Bagasse (**Figure 1C**). However, the cross-section and longitudinal section figure of the root system from treatment DI (**Figure 1D**) were filled with fungal mycelium. In addition, it also shown individual roots are being killed and fully infected by the invading fungus of *D. indusiata*. Approximately 5 g of healthy (disease-free) root sample was collected from each plant, washed three times with sterile water, immediately frozen in liquid nitrogen, and stored at –80°C for further analysis.

### 2.2 Methods

The progression of this study follows the technical route shown in **Figure 1E**.

#### 2.2.1 Method for calculating sugarcane yields

We chose an area of about 660 square meters to conduct sugarcane yield detection. Through statistics, we found a total of 4320 sugarcane plants were cultivated, with an average of 288 plants per row for 15 rows. The Plant height and Stem diameter were directly measured in meters. For plant height, the length from the first node above ground to the top node with an internode length of at least 2 cm was measured. Stem diameter was measured at the sixth node from the ground. The “Single stem weight” was obtained by removing the leaves from the sugarcane stem and then measuring the weight. For the Effective Stem of per-are calculation, 15 effective stems (over 1 meter in height) were randomly selected from each treatment in each row of sugarcane, and the average value was calculated and then totaled for all rows. The Yield of per-are (about 660 m^2^) was obtained by multiplying the Effective Stem data by the Effective Stem of per-are data.

#### 2.2.2 Metabarcoding

DNA was extracted from the leaf and root samples using the CTAB method (Cheng et al., 2003). Subsequently, the bacterial 16S rRNA V5–V7 region was amplified with the 799F and 1193R primers (Beckers et al., 2016), while the fungal ITS 1–2 region was amplified with the ITS1-F and ITS2 primers (Manzar et al., 2022). The amplification process involved a reaction mixture containing 1× Phusion^®^ High-Fidelity PCR Master Mix (New England Biolabs), 0.2 µM of forward and reverse primers, and approximately 10 ng of template DNA. The thermal cycling conditions included an initial denaturation at 98°C for 1 min, followed by 30 cycles of denaturation at 98°C for 10 s, annealing at 50°C for 30 s, elongation at 72°C for 30 s, and a final extension at 72°C for 5 min. Subsequently, sequencing libraries were generated using the TruSeq^®^ DNA PCR-Free Sample Preparation Kit (Illumina, USA) as per the manufacturer’s recommendations, and index codes were added. The library quality was assessed using a Qubit@ 2.0 Fluorometer (Thermo Scientific) and an Agilent Bioanalyzer 2100 system. The purified amplicon libraries were then pooled in equimolar ratios and subjected to paired-end sequencing (PE250) on an Illumina platform (Novaseq 6000 sequencing) following the standard protocol (Second generation sequencing). For a detailed classification of the endophytic bacteria in the root system, the full-length 16S rDNA was amplified from root samples in the DI treatment using the 27F and 1492 R primers (Kesharwani et al., 2022). Subsequently, sequencing libraries were generated using the SMRTbell TM Template Prep Kit (PacBio, Menlo Park, CA, USA) as per the manufacturer’s recommendation. These libraries were then sequenced on a PacBio Sequel platform (Third generation sequencing).

The data were analyzed using Kujawska’s approach (Kujawska et al., 2021). Initially, the paired-end reads were assembled and subjected to quality control. High-quality reads were assigned to samples based on their unique barcode, and then truncated by removing the barcode and primer sequence. Subsequently, FLASH (V1.2.7) was used to merge the paired-end reads and generate raw tags (Magoč and Salzberg, 2011). These raw tags underwent quality filtering under specific conditions to obtain high-quality clean tags (Bokulich et al., 2013), following the QIIME (v1.9.1) quality control process (Caporaso et al., 2010). The resulting tags were compared with the SILVA database (v132) (16S rRNA metabarcoding data) (Pruesse et al., 2007) and the UNITE database (v8.0) (ITS metabarcoding data) (Nilsson et al., 2019) using the UCHIME algorithm (Edgar et al., 2011) to detect and remove chimera sequences (Haas et al., 2011), resulting in the acquisition of effective tags. The relative abundance of each operational taxonomic unit (OTU) and taxonomic grouping (e.g., Genus and Family levels) were calculated by counting the number of tags and expressing it as a percentage. For the full-length 16S rRNA sequencing reads, the DADA2 R package (Callahan et al., 2016) (v1.14) was utilized to implement a complete pipeline for denoising and removing chimeras, resulting in inferred sample sequences. Subsequently, the reads were clustered into OTUs using the UPARSE software (v7.0.1001) (Edgar, 2013), with sequences having ≥97% similarity being assigned to the same OTUs. The representative sequences from each OTU were then assigned to bacterial and fungal taxa based on the SILVA (v132) and the UNITE (v8.0) databases, using a confidence threshold value of 0.8.

Additionally, alpha diversity indices, including observed species richness (Sobs), Chao1, and Shannon indices, were utilized to evaluate the species complexity in the samples. These indices were calculated using QIIME software (v1.9.1) and visualized using R software (v2.15.3). Principal component analysis (PCA) based on the OTU number was performed to assess the similarity among the samples using the R project vegan package (v2.6-2; http://CRAN.R-project.org/package=vegan; 2022.4.20) and the ggplot2 package (v3.3.3; http://CRAN.R-project.org/package=ggplot2; 2022.3.1). Furthermore, Venn analysis was conducted to compare the OTUs of different groups using the VennDiagram package (v1.6.16) in R (Chen and Boutros, 2011). These analyses were carried out using the OTU numbers without any model transformation.

For the full-length 16S rRNA sequences, taxonomic classification was performed using BLAST (v2.6.0) (Altschul et al., 1990). The representative OTU sequences or ASV sequences were searched against the NCBI 16S ribosomal RNA Database (Bacteria and archaea) (http://www.ncbi.nlm.nih.gov) (v202101) using the best hit criteria (E-value < e-5, query coverage ≥ 60%; sequence identity ≥92% was considered to belong to the same species; sequence identity ≥88% was considered to belong to the same genus; sequence identity ≥85% was considered to belong to the same family; sequence identity ≥80% was considered to belong to the same order; classes were inferred when sequence identity ≥75%; and phylum were inferred when sequence identity ≥70%).

#### 2.2.3 Widely targeted metabolome analysis

A comprehensive metabolome analysis using ultra-high-performance liquid chromatography-electrospray ionization-tandem mass spectrometry (UHPLC-ESI-MS/MS) was conducted at Metware Biotechnology Co., Ltd. (Wuhan, China) to identify differences in metabolites among the root samples, as previously described (Duan et al., 2022b). The root samples underwent freeze-drying for 48 hours and were ground into powder. Approximately 100 mg of the powder was then extracted with 70% aqueous methanol (0.6 mL) and analyzed using a UHPLC-ESI-MS/MS system (UHPLC, Shim-pack UFLC SHIMADZU CBM30A system, Kyoto, Japan; MS, Applied Biosystems 4500 Q TRAP, Framingham, MA, USA). Each root sample had three biological replicates, and their extracts were combined in equal amounts to create a quality control (QC) sample for assessing measurement accuracy after every six samples.

The qualitative analysis of the primary and secondary mass spectrometry data was performed using a self-built database, MWDB (v2.0; Metware Biotechnology Co., Ltd. Wuhan, China), as well as publicly available databases such as MassBank (http://www.massbank.jp), HMDB (Human Metabolome Database; http://www.hmdb.ca), and METLIN (http://metlin.scripps.edu/index.php). Additionally, the quantitative analysis of the metabolites was carried out using the multiple reaction monitoring mode (MRM) of triple quadrupole mass spectrometry. The MultiQuant software (v3.0.2) was employed to access the mass spectrometry files, integrate and correct the peaks, with the chromatographic peak area representing the relative content of the metabolite.

The raw metabolite data were processed using Analyst 1.6.3 software (AB Sciex, Framingham, MA, USA). The original metabolite abundance was log-transformed to reduce variance and normalize the data. Subsequently, PCA, cluster analysis, and orthogonal projections to latent structures-discriminant analysis (OPLS-DA) were conducted with the metabolite data in R (http://www.r-project.org/) following previously described methods (Li et al., 2021). VIP values of all metabolites from the OPLS-DA were extracted using the first component. Finally, metabolites from various pairwise comparisons (CK vs. Bagasse, CK vs. DI, and Bagasse vs. DI) with VIP ≥ 1 (indicating high confidence in pairwise comparisons) and a fold change ≥ 2 and ≤ 0.5 were identified as differential metabolites. Kyoto Encyclopedia of Genes and Genomes (KEGG) annotation and metabolic pathway analysis were performed for the differential metabolites using a hypergeometric test to identify significantly enriched pathways (p < 0.05).

#### 2.2.4 Correlation analysis

Finally, the correlation between the metabarcoding and metabolome data was analyzed using OmicShare tools, a free online platform for data analysis (https://www.omicshare.com/tools). The Z-score (zero-mean normalization; z = (x - µ)/σ, where x is the original value, µ is the mean value, and σ is the standard deviation value) of the marker metabolites (peak area units) and the OTU numbers of the top ten fungal and bacterial genera with the highest relative abundance in the root samples were utilized to assess the correlation. Subsequently, a heat map was generated using the Pearson correlation coefficients with the correlation heat map tools in OmicShare.

## 3 Results

### 3.1 Sugarcane yield statistics

The study first analyzed the yield of sugarcane under CK, Bagasse and DI treatments. After 11 months of growth, the sugarcane from DI group showed a significant yield advantage (**Table 1**). We conducted statistical analysis on five agronomic traits of plant height (cm), stem diameter (cm), effective Stem of per-are, single stem weight (kg) and yield of per-are (kg). The DI group showed advantage in plant height (241.67 cm, 250.27 cm, and 254.94 cm in CK, Bagasse, and DI), effective stem of per-are (3973, 3672, and 4540), single stem weight (1.27 kg, 1.41 kg and 1.40 kg), and yield of per-are (5045.71 kg, 5177.52 kg, and 6356 kg) compared with CK and Bagasse. These observations suggest that DI could significantly improve the yield by increasing the tillering of sugarcane compared with Bagasse.

**Table 1:** Sugarcane yield statistics.

	CK	Bagasse	DI
Plant height (cm)	241.67	250.27	254.94
Stem diameter (cm)	2.55	2.63	2.57
Effective Stem of per-are (~ 660 m²= 1 are)	3973	3672	4540
Single stem weight (kg)	1.27	1.41	1.4
Yield of per-are (kg)	5045.71	5177.52	6356

Plant height (cm), stem diameter (cm), and single stem weight (kg) are based on 15 replicates; the effective stem of per-are and yield of per-are were determined based on the conversion estimate. Yield of per-are = Single stem weight * Effective Stem of per-are. All values represent the mean values.

### 3.2 Microbial diversity

#### 3.2.1 Metabarcoding

**Figure 2:**
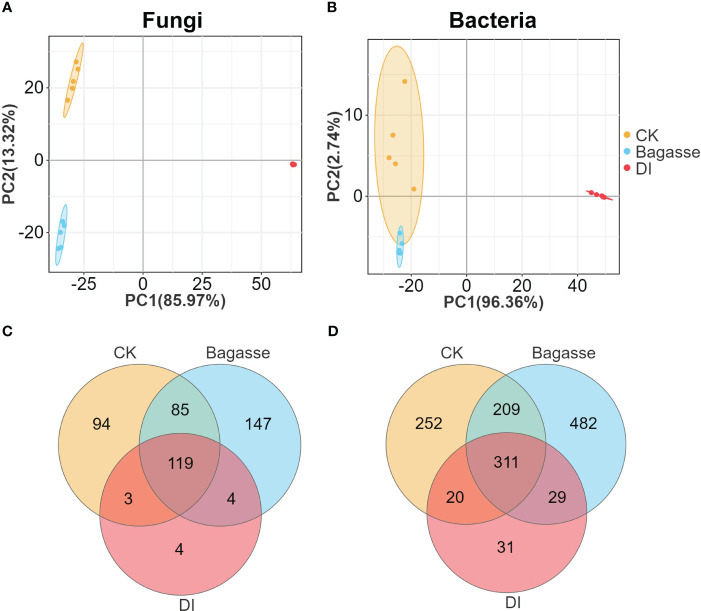
PCA and Venn analysis of the endophytic OTUs in sugarcane roots under CK, Bagasse, and DI. PCA of the **(A)** bacterial and **(B)** fungal OTUs. Venn analysis of the **(C)** fungal and **(D)** bacterial OTUs.

We performed metabarcoding sequencing using the root system from three treatments to reveal how the “white root” phenotype induced by *D. indusiata* affects the endophytic ecology of sugarcane roots. A total of 3,876,163 effective metabarcoding tags were obtained from 15 samples via sequencing of the fungal ITS 1–2 region and the bacterial 16S rRNA V5–V7 region. Subsequent clustering identified an average of 226 fungal OTUs (ITS) and 598 bacterial OTUs (16S rDNA) per sample (**Supplementary Table S1**). The PCA scatter plots based on the fungal and bacterial OTU abundance in the roots showed clear separation of CK, Bagasse, and DI samples, which indicates that both the process of sugarcane bagasse (Bagasse treatment) and the cultivation of DI (DI treatment) have the ability to regulate the composition of the root endophytic microbial community; here, PC1 contributed to 85.97% and 96.93% variations in fungi and bacteria (**Figures 2A, B**). The Venn diagram showed that 119 and 311 fungal and bacterial OTUs were shared among all root samples. Meanwhile, DI had the least unique fungal OTUs (94, 147, and 4 in CK, Bagasse, and DI; **Figure 2C** and bacterial OTUs (252, 482, and 31; **Figure 2D**). These observations suggest that *D. indusiata* had a drastic synergistic effect on sugarcane root endophytic fungi and bacteria.

#### 3.2.2 Microbial alpha diversity and key microbial taxa

**Figure 3:**
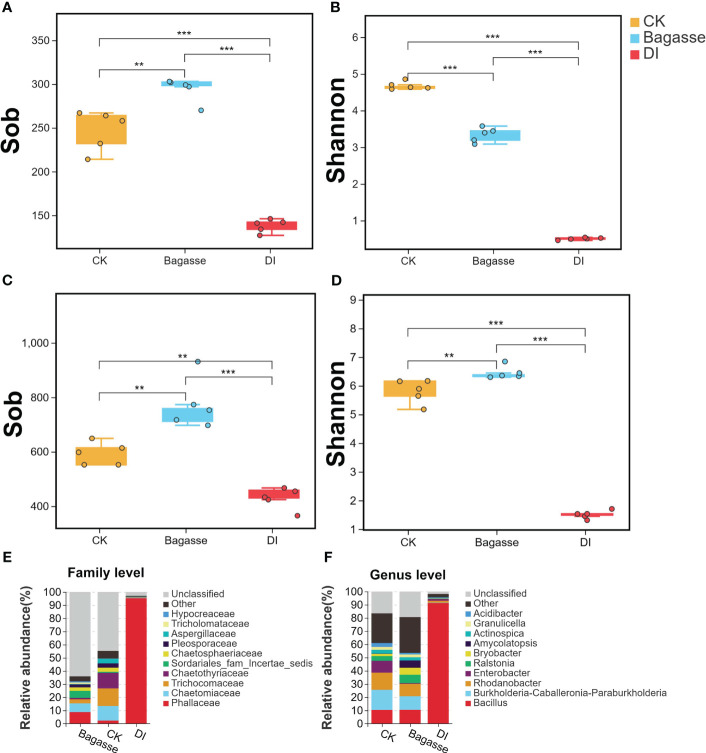
Alpha diversity and community composition of microbes in sugarcane root under CK, Bagasse, and DI. **(A–D)** Boxplot shows the alpha diversity indices of the endophytic fungi and bacteria in the sugarcane roots. Observed species index (Sob) of **(A)** fungi and **(C)** bacteria. Shannon index of **(B)** fungi and **(D)** bacteria. All indices were calculated based on OTU. The top and bottom whiskers of the boxes represent the maximum and minimum values; the line inside the box represents the median, the top margin of the box represents the upper quartile, and the lower margin of the box represents the lower quartile. The scatters represent repetition (N = 5). “**” and “***” indicate significance at *P* < 0.01 and *P* < 0.001, respectively. **(E, F)** The relative abundance of fungal community at the family level **(E)** and bacterial community at the genus level **(F)** in the sugarcane roots.

We further calculated the alpha diversity indices to determine the species diversity and used the *t*-test to compare the samples. Sobs for root samples of CK, Bagasse, and DI ranged from 127 to 303 for fungi (**Figure 3A**) and 365 to 931 for bacteria (**Figure 3C**). Meanwhile, the Shannon index ranged from 0.46 to 4.85 for fungi (**Figure 3B**) and 1.31 to 6.84 for bacteria (**Figure 3D**). Besides, both Sobs and Shannon index were significantly different among CK, Bagasse, and DI. Among them, the largest difference was detected in the CK vs. DI and Bagasse vs. DI comparisons (*P* < 0.01, **Figure 3A–D**), which DI shown the significantly lowest diversity, regardless of whether it was measured by Sobs and Shannon index (e.g., bacterial Shannon index CK vs. DI was 1.5 vs. 6.45; **Figure 3D**). In addition, other alpha indices, such as simpson, chao, and ace, have also exhibited similar trends to the Shannon index. (detailed information shown in **Supplementary Table S2**). These observations suggest that the diversity of root fungi and bacteria decreased dramatically under DI compared with CK and Bagasse.

Further taxonomic annotation based on the SILVA and UNITE databases revealed that the differences in the endophytic fungi and bacteria were responsible for the huge differences in alpha diversity indices between the treatments. As shown in **Figure 3E**, Phallaceae (2.36%, 8.82%, and 95.28% in CK, Bagasse, and DI groups), Chaetomiaceae (11.07%, 6.63%, and 0.47%), and Trichocomaceae (13.34%, 3.02% and 0.22%) were the top three most abundant fungi at the family level. Meanwhile, *Bacillus* (10.4%, 10.47%, and 91.32%), *Burkholderia-Caballeronia-Paraburkholderia* (15.2%, 10.31%, and 0.35%), and *Rhodanobacter* (12.99%, 9.36% and 1.35%) were the top three most abundant bacteria at the genus level (**Figure 3F**). Above shown in DI treatment, Phallaceae and *Bacillus* were high compared with CK and Bagasse; The abundance of Chaetomiaceae, Trichocomaceae, *Burkholderia-Caballeronia-Paraburkholderia* and *Rhodanobacter* decreased after Bagasse and DI treatment.

#### 3.2.3 ITS-16S rRNA correlation analysis

**Figure 4:**
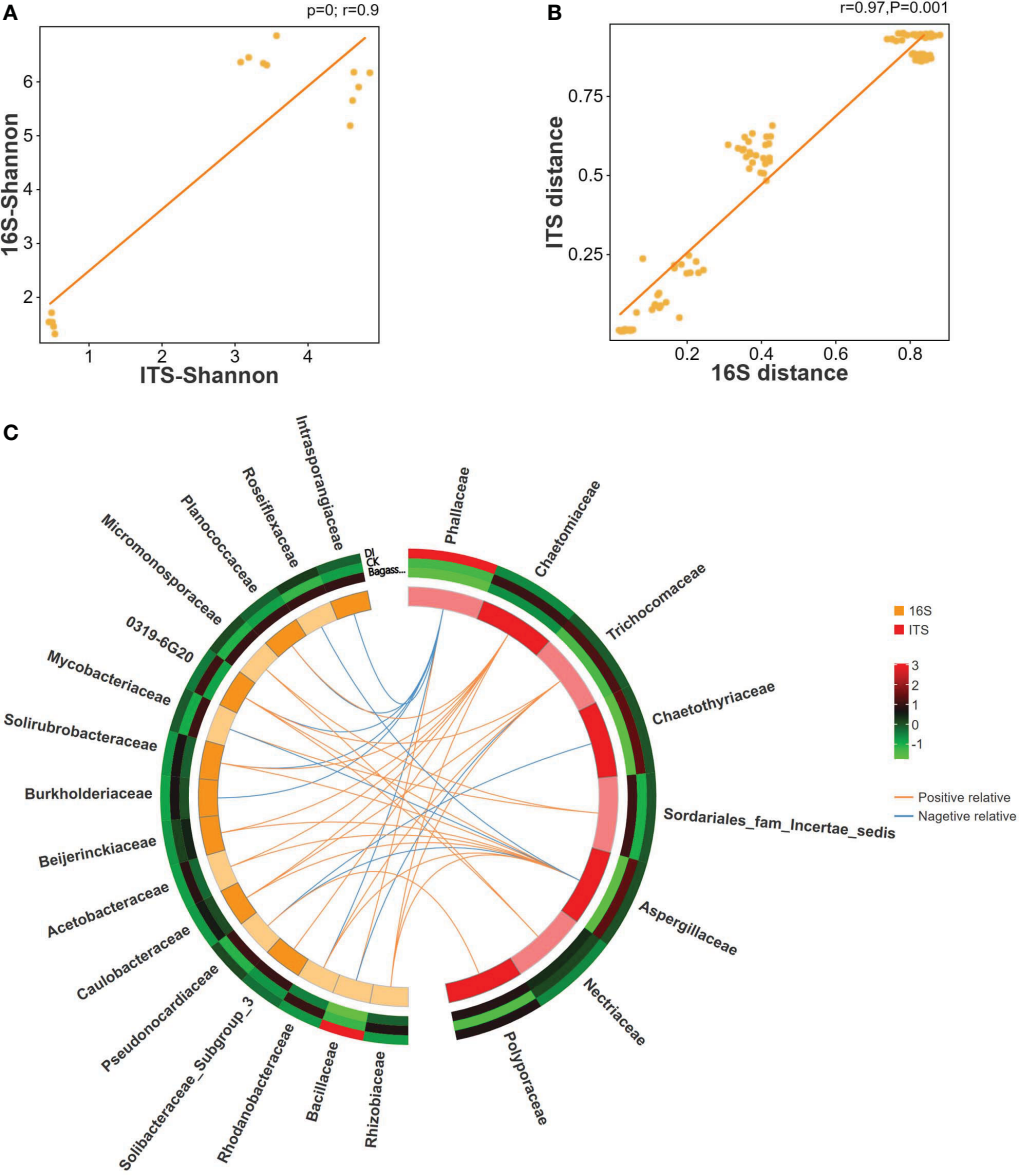
Correlation between ITS and 16S rRNA of sugarcane root under CK, Bagasse, and DI. **(A)** Pearson correlation analysis of Shannon index based on ITS and 16S rRNA OTUs. The vertical and horizontal axes show the coefficients from the Shannon index of the samples in ITS and 16S rRNA OTUs. The indicator line represents the linear correlation coefficient of the fit, and the dots represent samples. **(B)** Mantel test based on Bray-Curtis distance matrix. The horizontal axis represents the 16S rRNA distance matrix, and the vertical axis represents the ITS distance matrix. A dot in the figure represents a pair of samples. The indicator line represents the linear correlation coefficient of the fit. **(C)** Circos map of the sPLS model shows the correlation between fungi and bacteria. The outside circle external heat map shows the abundance of the microbial families under different treatments, and the color represents abundance while the red represents higher abundance and green represents lower abundance. In the internal Circos map, the colors represent fungi and bacteria, each cell represents a family, the lines represent correlations, and the orange line represents a positive correlation and the blue line represents a negative correlation.

To further reveal the consistency in diversity of root fungi and bacteria under CK, Bagasse, and DI, which to investigate whether there is any interaction between root-associated fungi and bacteria in terms of endophytic colonization, we performed ITS-16S rRNA association analysis (Including Pearson correlation analysis, sPLS (sparse partial least squares) association analysis). The Pearson correlation coefficients and Mantel test based on the Shannon index from alpha diversity (R-value = 0.9, **Figure 4A**) and genetic distance from beta diversity (R-value = 0.97, **Figure 4B**) showed significant correlations between fungi and bacteria. Further sPLS association analysis was conducted based on the OTU data of fungi and bacteria (family level) with R-values greater than 0.5 or less than -0.5. While Phallaceae showed a strong positive correlation with Bacillaceae (R = 0.98) and negative correlations with many other bacterial families, such as Burkholderiaceae (R = -0.98) and Rhodanobacteraceae (R = -0.93) (**Figure 4C**). Meanwhile, Bacillaceae showed the trend of passive regulation, including only a negative association with Trichocomaceae (R = -0.58). These observations indicate a specific pattern of fungi interacting with bacteria, that the root invasion of *D. indusiata* (Family: Phallaceae) leads to a large-scale colonization of Bacillaceae in sugarcane root system.

#### 3.2.4 Full-length 16S rRNA sequencing of roots under DI

To further reveal which bacterial species of the Bacillaceae family were promoted by *D. indusiata* invasion to the root during DI, we performed third-generation sequencing (PACBIO) and generated the full-length 16S rRNA of roots. A total of 63656 clean CCS (Circular Consensus Sequence) reads were obtained as full-length reads, with an L50 size of 1528 bp and L90 size of 1512 bp. Then, 505 ASVs (amplicon sequence variants) were obtained from one DI root sample after clustering analysis. Further analysis of the top 10 ASVs with the highest relative abundance (**Table 2**) revealed that 9 out of 10 belonged to Bacillaceae, including *Bacillus aryabhattai* (top 1, 4, 7, and 10 with relative abundance at 27.39%, 3.63%, 2.92%, and 1.49%), *Priestia megaterium* (top 2, 3 and 5, with 15.93%, 4.52% and 3.51%), *Bacillus cereus* (top 6 with 3.27%), and *Bacillus altitudinis* (top 8 with 2.42%). These observations suggest that *D. indusiata* could cause the colonization of *B. aryabhattai* and *Priestia megaterium*, from the Bacillaceae family, in the roots of sugarcane; *Bacillus aryabhattai* was the main bacterial species (total relative abundance of 35.43%).

### 3.3 Study on the metabolome of sugarcane root system

#### 3.3.1 Metabolite composition

**Figure 5:**
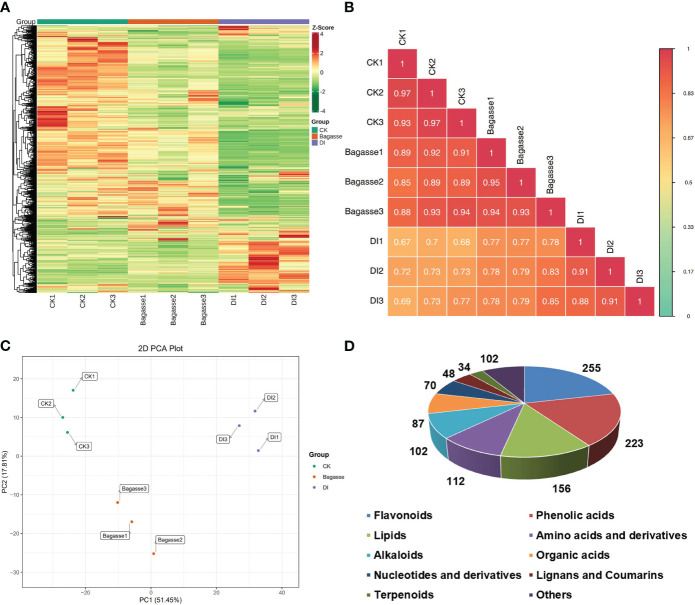
Metabolic composition of sugarcane roots under CK, Bagasse, and DI. **(A)** Clustering heat map of all metabolites. Each column represents a sample, and each row represents a metabolite. The color bar represents the metabolite abundance. Different shades of red and green represent the upregulated and downregulated metabolites, respectively. **(B)** Pearson’s correlation heat map between CK, Bagasse, and DI. The longitudinal and diagonal lines show the sample names and different colors represent the Pearson correlation coefficients; the key is shown on the right side. **(C)** PCA plot based on metabolite peak area unit of root samples. PC1 represents the first principal component, and PC2 represents the second principal component. The percentage represents the interpretation rate of the principal component of the data set from peak area unit. A dot represents a sample, and samples in the same color are of the same group. **(D)** Pie figure shows the proportion classes of metabolites. The number near each metabolite class represents metabolite number.

The present study related the regulation of root endophytic diversity by the “white root” phenotype induced by *D. indusiata* to its yield enhancement. Therefore, we conducted a widely targeted metbaolome analysis (UPLC-ESIMS/MS) on the sugarcane roots from CK, Bagasse, and DI treatments. The heat map based on the abundance of metabolites (**Figure 5A**) showed large-scale variance for the DI group compared with CK and Bagasse, which indicated the “white root” greatly affected root metabolism. PCA based on metabolite peak area unit showed a clear separation of CK, Bagasse, and DI groups (**Figure 5C**), which indicates that both the process of Bagasse and DI treatment could regulate the metabolite composition of sugarcane roots. Then, the sample-to-sample correlation heat map based on metabolite further verified the above pattern (**Figure 5B**), and DI group showed a lower correlation than CK and Bagasse groups, which indicates that the cultivation of endophytic symbionts (DI treatment) has a significant impact on the metabolic processes and composition of metabolites in sugarcane roots. Specifically, based on public and commercial databases, 1189 metabolites (grouped into ten classes) were identified from these root samples under CK, Bagasse, and DI, including 255 flavonoids, 223 phenolic acids, 156 lipids, 112 amino acids and derivatives, 102 alkaloids, 97 organic acids, 70 nucleotides and derivatives, 48 lignans and coumarins, 34 terpenoids, and 102 others (**Figure 5D**). Detailed information on these metabolites is presented in **Supplementary Table S3**.

#### 3.3.2 Differential metabolites

**Figure 6:**
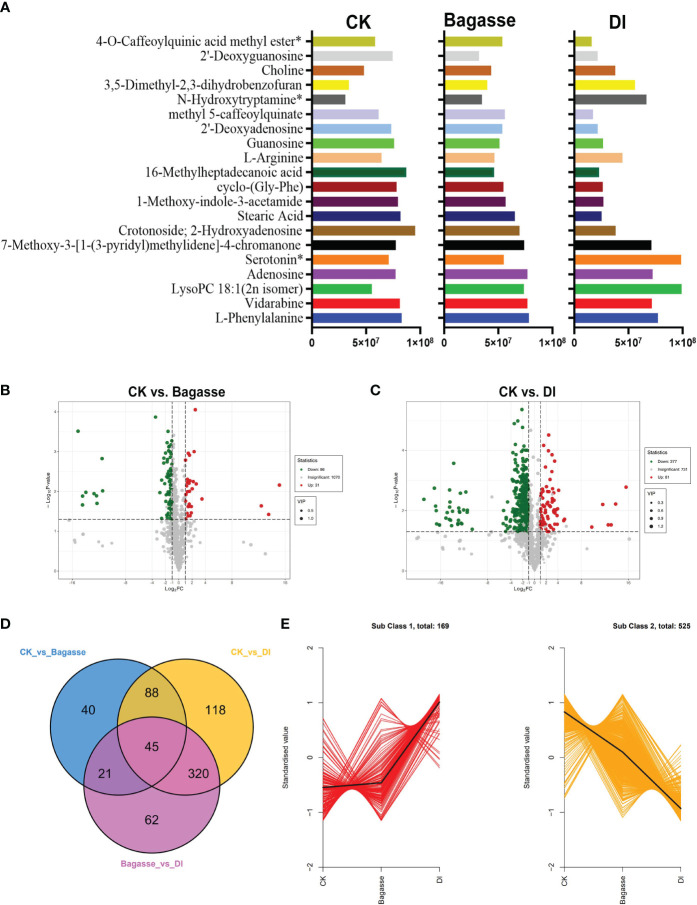
Differential metabolites of sugarcane root under CK, Bagasse, and DI. **(A)** Top 20 most abundant metabolites of sugarcane root under CK, Bagasse, and DI. The abscissa represents peak area units, and the ordinate represents the differential metabolites. **(B)** Volcano map of differential metabolites identified from the comparison groups CK vs. Bagasse, CK vs. DI. Each point in the volcano plot represents a metabolite; green represents the downregulated metabolites, red represents the upregulated metabolites, and gray represents the metabolites with no significant difference. The horizontal coordinate represents the log value of the metabolite difference between the comparison groups (log^2^FC); the ordinate represents the significance level (-log^10^ *P*-value); the dot size represents the VIP value. **(C)** Venn diagram shows the differential metabolites of different comparison groups. **(D)** K-means clustering of metabolites of sugarcane root under CK, Bagasse, and DI. The abscissa represents the sample group, the ordinate represents the standardized relative metabolite content, and the class represents the metabolite category number with the trend in sugarcane root under CK, Bagasse, and DI. *Indicates the metabolite presence of isomer.

Furthermore, we analyzed metabolites’ abundance and distribution characteristics in the root samples under CK, Bagasse, and DI. The analysis identified l-phenylalanine, vidarabine, lysopc 18:1(2n isomer), adenosine, serotonin*, 7-methoxy-3-[1-(3-pyridyl)methylidene]-4-chromanone, crotonoside; 2-hydroxyadenosine, stearic acid, 1-methoxy-indole-3-acetamide, cyclo-(gly-phe), 16-methylheptadecanoic acid, l-arginine, guanosine, 2’-deoxyadenosine, methyl 5-caffeoylquinate, n-hydroxytryptamine*, 3,5-dimethyl-2,3-dihydrobenzofuran, choline, 2’-deoxyguanosine and 4-o-caffeoylquinic acid methyl ester*3 as the top 20 most abundant metabolites (**Figure 6A**). Further, the quanitifcation of the peak area unit showed that the abundance of metabolites in DI differed greatly compared with CK and Bagasse, while the abundance was similar in CK and Bagasse. Mainly, the abundance of Stearic Acid, 1-Methoxy-indole-3-acetamide, cyclo-(Gly-Phe), and 16-Methylheptadecanoic acid in DI was different from that in Bagasse and CK (**Figure 6A**). Next, we analyzed the differential metabolites of the CK vs. Bagasse and CK vs. DI comparation groups. As shown in **Figures 6B, C**, 31 and 81 metabolites were upregulated, and 86 and 377 metabolites were downregulated in CK vs. Bagasse and CK vs. DI, respectively, which suggests that the “white root” phenotype (DI) greatly changed the root metabolism compared with the CK. Venn analysis showed that CK vs. DI (118) and Bagasse vs. DI (62) comparisons had more unique metabolites than CK vs. Bagasse (40) (**Figure 6D**). Then, we analyzed the distribution characteristics of all metabolites according to the K-means clustering method and identified two distinct metabolic clustering patterns. As shown in **Figure 6E** and **Supplementary Table S4**, the abundance of subclass 1, including 169 metabolites, was high in DI but similar in CK and Bagasse. Meanwhile, the abundance of subclass 2, including 525 metabolites, was the highest in CK and the lowest in DI. Our differential metabolite analysis thus indicated that the *D. indusiata*-induced “white root” phenotype greatly affected the metabolic process of sugarcane roots.

#### 3.3.3 Kyoto Encyclopedia of Genes and Genomes analysis

**Figure 7:**
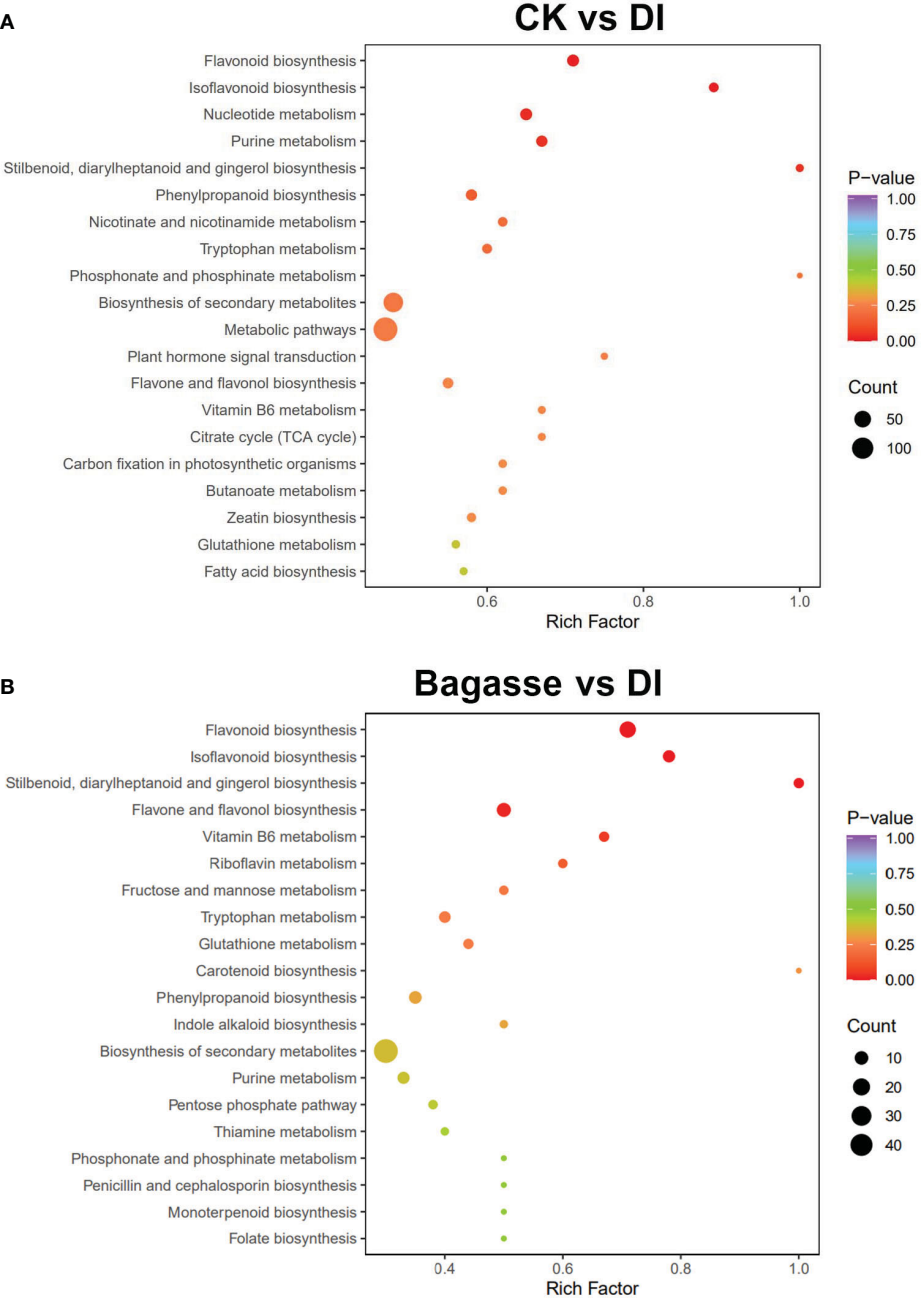
KEGG enrichment analysis of differential metabolites of sugarcane root under Bagasse and DI. **(A)** stands KEGG enrichment analysis of CK vs. DI, and **(B)** stands Bagasse vs. DI. Each bubble in the plot represents a metabolic pathway; the abscissa and the bubble size jointly indicate the magnitude of impact. A larger bubble indicates that more metabolites are enriched; the bubble color represents the *P*-value of the enrichment analysis, with darker colors indicating a higher degree of enrichment.

Further, we performed a KEGG enrichment analysis to determine the function of the differential metabolites regulated by the *D. indusiata*-induced “white root” phenotype. The differential metabolites identified from the CK vs. DI group significantly enriched (*P* < 0.05) “Flavonoid biosynthesis (ko00941)”, “Isoflavonoid biosynthesis (ko00943)”, “Nucleotide metabolism (ko01232)”, “Purine metabolism (ko00230)”, and “Stilbenoid, diarylheptanoid and gingerol biosynthesis (ko00945)” pathways (**Figure 7A**); The differential metabolites identified from the CK vs. DI group significantly enriched (*P* < 0.05) Flavonoid biosynthesis (ko00941), Isoflavonoid biosynthesis (ko00943), Stilbenoid, diarylheptanoid and gingerol biosynthesis (ko00945) and Flavone and flavonol biosynthesis (ko00944) pathways (**Figure 7B**). Detailed information on the metabolite enrichment is provided in the “kegg_map” part of **Supplementary Table S3**. The KEGG enrichment analysis indicated that flavonoid metabolism is an important pathway regulated by the “white root” phenotype discovered in this study.

#### 3.3.4 Association analysis of metabolites with fungi and bacteria

**Figure 8:**
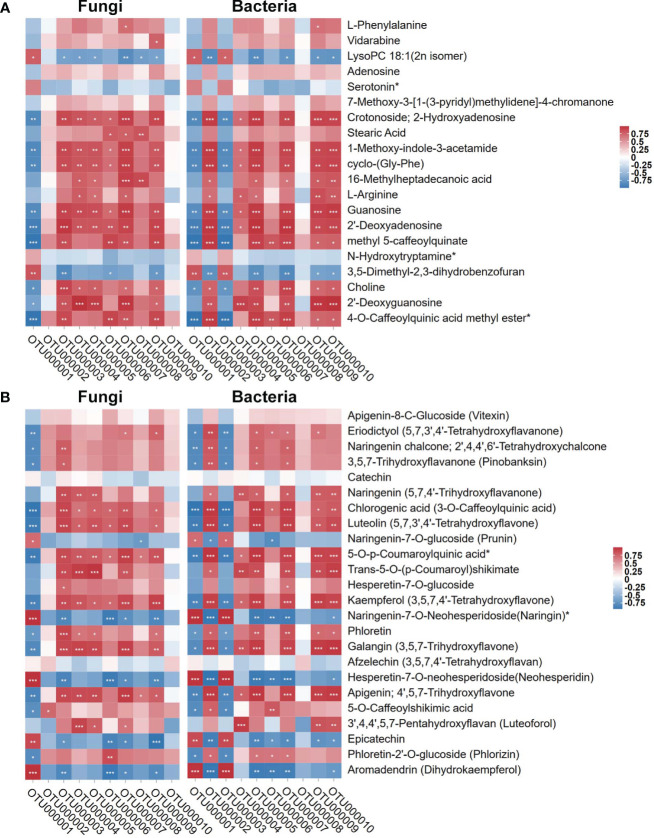
Pearson correlation of top 10 OTUs of endophytes (fungi and bacteria) with the top 20 most abundant metabolites and metabolites involved in “Flavonoid biosynthesis” KEGG pathway (ko00941). **(A)** stand the top 20 most abundant metabolites. **(B)** stand metabolites involved in “Flavonoid biosynthesis” KEGG pathway (ko00941). Red and blue represent Pearson correlation (*r*); the key is shown at the top right side. ‘*’, ‘**’, and ‘***’ indicate significant correlation at *P* ≤ 0.001, 0.001 < *P* < 0.01, respectively; not marked: insignificant correlation (0.01 < *P* < 0.05; *P* ≥ 0.05).

Since the regulatory trend of microbial diversity showed consistency with the root metabolome result, we analyzed root metabolites affected the DI-mediated structure and related *Bacillus association.* We used the top 10 fungal and bacterial OTUs with high relative abundance based on tag numbers to analyze the association with the significantly regulated metabolites (base peak area unit; the top 20 most abundant ones) in sugarcane roots under CK, Bagasse, and DI (**Figure 6A**) and metabolites associated with the “Flavonoid biosynthesis (ko00941)” pathway. As shown in **Figure 8**, according to the study’s microbial diversity results, out000001 from fungi and bacteria could stand for *D. indusiata* (Because *D. indusiata* belongs to Phallaceae family, which is the dominance abundance family of DI group, **Figure 3E**) and *B. aryabhattai* (**Table 2**), respectively. The analysis using the top 20 most abundant metabolites revealed that *D. indusiata* (OTU000001 of fungi) and *B. aryabhattai* (OTU000001 of bacteria) were positively correlated (*P* < 0.05) with lysopc 18:1(2n isomer) and 3,5-dimethyl-2,3-dihydrobenzofuran (r > 0.5) and negatively correlated with crotonoside, 2-hydroxyadenosine, 1-methoxy-indole-3-acetamide, cyclo-(gly-phe), guanosine, 2’-deoxyadenosine, methyl 5-caffeoylquinate, choline and 2’-deoxyguanosine (r < -0.5) (**Figure 8A**). Meanwhile, the analysis using metabolites associated with the “Flavonoid biosynthesis” (ko00941) pathway revealed that *D. indusiata* (OTU000001 of fungi) and *B. aryabhattai* (OTU000001 of bacteria) were positively correlated (*P* < 0.05) with naringenin-7-o-glucoside (prunin), naringenin-7-o-neohesperidoside(naringin)*, hesperetin-7-o-neohesperidoside(neohesperidin), epicatechin and aromadendrin (dihydrokaempferol) (r > 0.5); and negatively correlated with eriodictyol (5,7,3’,4’-tetrahydroxyflavanone), naringenin chalcone; 2’,4,4’,6’-tetrahydroxychalcone, 3,5,7-trihydroxyflavanone (pinobanksin), chlorogenic acid (3-o-caffeoylquinic acid), luteolin (5,7,3’,4’-tetrahydroxyflavone), 5-o-p-coumaroylquinic acid*, kaempferol (3,5,7,4’-tetrahydroxyflavone), phloretin, galangin (3,5,7-trihydroxyflavone), apigenin; 4’,5,7-trihydroxyflavone, 5-o-caffeoylshikimic acid and phloretin-2’-o-glucoside (phlorizin) (r < -0.5) (**Figure 8B**).

**Table 2:** Full-length 16S rRNA taxonomy in sugarcane roots under DI.

ASV ID	Family	Genus	Species	Number	Relative abundance(%)
ASV000001	Bacillaceae	Bacillus	Bacillus aryabhattai	14091	27.39
ASV000002	Bacillaceae	Priestia	Priestia megaterium	8198	15.93
ASV000003	Bacillaceae	Priestia	Priestia megaterium	2323	4.52
ASV000004	Bacillaceae	Bacillus	Bacillus aryabhattai	1869	3.63
ASV000005	Bacillaceae	Priestia	Priestia megaterium	1808	3.51
ASV000006	Bacillaceae	Bacillus	Bacillus cereus	1680	3.27
ASV000007	Bacillaceae	Bacillus	Bacillus aryabhattai	1500	2.92
ASV000008	Bacillaceae	Bacillus	Bacillus altitudinis	1243	2.42
ASV000009	Symphyonemataceae	–	–	1216	2.36
ASV000010	Bacillaceae	Bacillus	Bacillus aryabhattai	767	1.49

## 4 Discussion

The sugarcane-*D. indusiata* intercropping system is a cultivation model used to increase the economic added value and yield. Interestingly, we have discovered a “white root” phenotype, where endogenetic fungi and bacteria interact together in the root system, when plant sugarcane intercropped with *D. indusiata*. Plant endophytes play a significant role in regulating root development and facilitating nutrient solubilization and transportation (Verma et al., 2021; Santoyo et al., 2016). This cultivation method which cased “white root” phenotype, utilizing DI treatment to activate endophytes, has increased sugarcane yield and represents a new cultivation approach with the potential to enhance sugarcane production.

Further analysis revealed that the diversity of root endophytic bacteria/fungi was significantly regulated under the DI treatment. Basically, the diversity of endophytes in the roots of sugarcane intercropped with *D. indusiata* was dominance in root, which was reflected in the large principal component differences (PC1 in **Figure 2A**) and dramatically shrinking alpha diversity index (**Figures 3A, C**). Interestingly, in the sugarcane root of DI, the *Bacillus* genus was associated with the same abundance as *D. indusiata* (Phallaceae) (**Figures 3E, F**). Interestingly, based on my preliminary research, it was also found that the DI treatment has the ability to reduce soil fungal diversity, which aligns with the observed decreasing trend in endophytic fungal diversity in this study (Duan et al., 2023a). This indicates that the cultivation of *D. indusiata* may invasive and symbiotic growth with sugarcane root, which in turn may regulate the metabolism of sugarcane roots and ultimately impact sugarcane growth. In the identified root-enriched fungal families in sugarcane root, the enrichment of Phallaceae in the root-associated fungi of the DI treatment suggests that the fungi belonging to this family originate from the mycelium of DI, as DI itself belongs to Phallaceae (Melanda et al., 2021); Chaetomiaceae family have the ability to regulate plant activity (Zhang et al., 2021); Trichocomaceae family is a common group of endophytic fungi, which can be found in such as *Bauhinia galpinii* and *Cycas bifida* (Feitosa et al., 2016; Zheng et al., 2018); The genus of *Bacillus* genus can produce a variety of compounds that participate in plant disease control and promote plant growth (Miljaković et al., 2020; Kashyap et al., 2022; Kashyap et al., 2021); The genus of *Burkholderia-Caballeronia-Paraburkholderia* group has been found to possess the ability to degrade herbicides (Zhao et al., 2022), suggesting that it may have the potential to assist sugarcane root in resisting herbicide damage; The genus of *Rhodanobacter* enhances plant tolerance to salt-induced osmotic stress, according to a recent study (Lee et al., 2019). The endophytic fungi and bacteria mentioned above all have the potential to regulate the growth of sugarcane.

Moreover, the ITS-16S rRNA correlation analysis confirmed a significant association (**Figure 4C**) between families of Phallaceae (DI) and Bacillaceae (Genus of *Bacillus*), indicating that *D. indusiata* mediates the intracellular colonization of *Bacillus* in the sugarcane root system. *Bacillus aryabhattai* was further identified as the dominant species from the specific genus based on our full-length sequencing. Thus, we propose that during the interacting association between *D. indusiata* and sugarcane roots, *B. aryabhattai* would be plays a prominent role, which may be an important factor regulating sugarcane growth. *Bacillus aryabhattai*, as a plant growth-promoting rhizobacteria (PGPR), has been isolated from the plant rhizosphere soil and reported to synthesize auxin and help the host to resist oxidation and heavy metal toxicity (Ghosh et al., 2018; Park et al., 2017; Bhattacharyya et al., 2017; Lee et al., 2012). Therefore, the study suggests that *B. aryabhattai* may be related to the yield increase in sugarcane under DI. Further investigation of the interaction between *D. indusiata* and *B. aryabhattai* in sugarcane roots will help promote sugarcane growth.

Among the metabolites identified with the highest peak areas in the Top 20, some have been reported to have the ability to affect plant growth. E.g., l-phenylalanine is a plant growth regulator capable of modulating phenolic compounds and enzymatic activity in sweet basil (Koca and Karaman, 2015); Vidarabine having antimicrobial actions, found in the *S. birrea* stem (bark) (Abdallah et al., 2021); Lysopc 18:1 has been reported to possess antipathogenic capabilities in tobacco plants (Wi et al., 2014); serotonin regulates plant growth functions, including roles in chronoregulation and the modulation of reproductive development (Erland et al., 2015). These high-abundance metabolites possess various functions in regulating plant growth and may be subject to regulation by the DI treatment, which in turn could influence the metabolic processes in the sugarcane root system.

The interaction between microorganisms and plant root systems typically triggers an immune response in the host plant (Berendsen et al., 2012), enhancing host resistance to stress (Castrillo et al., 2017), stimulating synthesis of beneficial metabolites, such as auxin, and thereby promoting host plant growth (Spaepen et al., 2007). In this study’s intercropping system, *D. indusiata* significantly improved the metabolism of sugarcane roots through root symbiosis. Studies have found that the *D. indusiata* fruiting bodies synthesize IAA (Duan et al., 2023b), and *D. indusiata* improves soil metabolism, accumulating carbohydrate metabolites (Duan et al., 2023a). These earlier reports suggested that *D. indusiata* promotes plant growth metabolism. In addition, the present study found that the interaction between *D. indusiata* and *B. aryabhattai* activates flavonoid metabolism in sugarcane roots. Flavonoids are natural polyphenols abundant in plants and play essential roles in biological processes within the plant and in response to environmental factors (Shen et al., 2022; Anjali et al., 2023). This change may be one of the important factors contributing to the increased tillering and yield in sugarcane under DI treatment. Earlier studies demonstrated that the wild mushroom *Leucocalocybe mongolica* similarly enhanced the flavonoid metabolism in *Leymus chinensis*, through soil transformation in a fairy ring ecosystem (Duan et al., 2022b; Duan et al., 2022a). Therefore, it is worth mentioning that the enrichment of certain metabolites is induced by the interaction between *D. indusiata* and *B. aryabhattai* to root. Among the various metabolites detected, lysoPC 18:1 is a naturally occurring bioactive lipid that plays a key role in the defense response of plants against pathogens (Wi et al., 2014). Besides, a study has reported that dihydrobenzofuran promotes plant growth by inhibiting IAA oxidase (Lee, 1976). Moreover, in the sugarcane root, various flavonoids were found enriched in response to the symbiotic stimulation of *D. indusiata* and *B. aryabhattai*, such as Naringenin-7-O-glucoside (Prunin), Naringenin-7-O-Neohesperidoside (Naringin)*, Hesperetin-7-O-neohesperidoside (Neohesperidin), Epicatechin, and Aromadendrin (Dihydrokaempferol), which are involved in the flavonoid biosynthesis (ko00941) pathway. These metabolites may be part of the mechanism by which *D. indusiata* stimulates flavonoid metabolism in sugarcane root and promotes growth and yield. However, further validation is required to provide a deeper understanding of the mechanisms by which *D. indusiata* interacts with sugarcane.

## 5 Conclusion

This study reports an interesting phenomenon where *D. indusiata* forms a “white root” phenotype with sugarcane and positively impacts sugarcane growth and yield in an intercropping system. Detailed analysis revealed that *D. indusiata* promotes flavonoid synthesis in sugarcane roots. The study provides new insights into using *D. indusiata* as a soil inoculant for plant growth promotion and a new approach for sugarcane growth study. However, further studies are required to identify the formation mechanism of this “white root” phenotype and determine the role of increased flavonoid synthesis in promoting sugarcane growth.

## Data availability statement

The datasets generated for this study upload in the NCBI Sequence Read Archive with submission of PRJNA1064011.

## Author contributions

MD: Conceptualization, Investigation, Software, Writing – original draft, Writing – review & editing. XL: Investigation, Software, Writing – review & editing. XW: Investigation, Writing – review & editing. SL: Investigation, Writing – review & editing. HH: Investigation, Validation, Writing – review & editing. YL: Investigation, Writing – review & editing. QL: Investigation, Writing – review & editing. GZ: Investigation, Writing – review & editing. BF: Investigation, Writing – review & editing. SQ: Investigation, Writing – review & editing. CL: Investigation, Writing – review & editing. HY: Investigation, Writing – review & editing. JQ: Investigation, Writing – review & editing. ZC: Conceptualization, Writing – review & editing. ZW: Conceptualization, Writing – review & editing.
